# Geotemporal Analysis of *Neisseria meningitidis* Clones in the United States: 2000–2005

**DOI:** 10.1371/journal.pone.0082048

**Published:** 2013-12-12

**Authors:** Ann E. Wiringa, Kathleen A. Shutt, Jane W. Marsh, Amanda C. Cohn, Nancy E. Messonnier, Shelley M. Zansky, Susan Petit, Monica M. Farley, Ken Gershman, Ruth Lynfield, Arthur Reingold, William Schaffner, Jamie Thompson, Shawn T. Brown, Bruce Y. Lee, Lee H. Harrison

**Affiliations:** 1 Department of Epidemiology, University of Pittsburgh Graduate School of Public Health, Pittsburgh, Pennsylvania, United States of America; 2 Infectious Diseases Epidemiology Research Unit, University of Pittsburgh Graduate School of Public Health and School of Medicine, Pittsburgh, Pennsylvania, United States of America; 3 Meningitis and Vaccine Preventable Diseases Branch, Division of Bacterial Diseases, Centers for Disease Control and Prevention, Atlanta, Georgia, United States of America; 4 New York State Department of Health, Albany, New York, United States of America; 5 Connecticut Department of Public Health, Hartford, Connecticut, United States of America; 6 Emory University and VA Medical Center, Atlanta, Georgia, United States of America; 7 Colorado Department of Public Health and Environment, Denver, Colorado, United States of America; 8 Minnesota Department of Health, St. Paul, Minnesota, United States of America; 9 University of California, Berkeley, Berkeley, California, United States of America; 10 Vanderbilt University School of Medicine, Nashville, Tennessee, United States of America; 11 Oregon Public Health Division, Portland, Oregon, United States of America; 12 Pittsburgh Supercomputing Center, Carnegie Mellon University, Pittsburgh, Pennsylvania, United States of America; 13 Public Health Computational and Operations Research (PHICOR), University of Pittsburgh School of Medicine and Graduate School of Public Health, Pittsburgh, Pennsylvania, United States of America; 14 Department of International Health, Johns Hopkins Bloomberg School of Public Health, Baltimore, Maryland, United States of America; Charité-University Medicine Berlin, Germany

## Abstract

**Background:**

The detection of meningococcal outbreaks relies on serogrouping and epidemiologic definitions. Advances in molecular epidemiology have improved the ability to distinguish unique *Neisseria meningitidis* strains, enabling the classification of isolates into clones. Around 98% of meningococcal cases in the United States are believed to be sporadic.

**Methods:**

Meningococcal isolates from 9 Active Bacterial Core surveillance sites throughout the United States from 2000 through 2005 were classified according to serogroup, multilocus sequence typing, and outer membrane protein (*porA*, *porB*, and *fetA*) genotyping. Clones were defined as isolates that were indistinguishable according to this characterization. Case data were aggregated to the census tract level and all non-singleton clones were assessed for non-random spatial and temporal clustering using retrospective space-time analyses with a discrete Poisson probability model.

**Results:**

Among 1,062 geocoded cases with available isolates, 438 unique clones were identified, 78 of which had ≥2 isolates. 702 cases were attributable to non-singleton clones, accounting for 66.0% of all geocoded cases. 32 statistically significant clusters comprised of 107 cases (10.1% of all geocoded cases) were identified. Clusters had the following attributes: included 2 to 11 cases; 1 day to 33 months duration; radius of 0 to 61.7 km; and attack rate of 0.7 to 57.8 cases per 100,000 population. Serogroups represented among the clusters were: B (n = 12 clusters, 45 cases), C (n = 11 clusters, 27 cases), and Y (n = 9 clusters, 35 cases); 20 clusters (62.5%) were caused by serogroups represented in meningococcal vaccines that are commercially available in the United States.

**Conclusions:**

Around 10% of meningococcal disease cases in the U.S. could be assigned to a geotemporal cluster. Molecular characterization of isolates, combined with geotemporal analysis, is a useful tool for understanding the spread of virulent meningococcal clones and patterns of transmission in populations.

## Introduction

*Neisseria meningitidis* is an important cause of meningitis and other serious bacterial infections globally. [1] In the U.S., over 98% of meningococcal disease cases are considered to be sporadic, that is, unrelated to other cases, but outbreaks also occur. [2], [3] Identification of meningococcal outbreaks typically relies on serogrouping and epidemiologic definitions. [3], [4] The incidence of meningococcal disease in the U.S. is at historically low levels [2].

Advances in objective, DNA sequence-based molecular epidemiologic tools for *N. meningitidis* have enhanced the ability to characterize this organism. Multilocus sequence typing (MLST) is a standard molecular subtyping approach for determining genetic lineage. [5] DNA sequencing of genes that encode outer membrane proteins (OMPs) provides additional discriminatory power among strains belonging to the same sequence type (STs). [6], [7] Together, MLST and OMP genotyping allow for the classification of meningococcal isolates into specific clones, which can be used to detect outbreaks and study patterns of spread within populations. [8]–[10] In one study a spatial scan statistic was used to identify 26 clusters of invasive meningococcal disease in Germany using a clone definition based on serogroup, *por*A and *fet*A typing [8].

We recently reported the population structure of invasive meningococcal isolates throughout the United States. [11] The purpose of the present study was to assess geotemporal clustering patterns of specific meningococcal clones among the isolates reported in that study to determine whether this approach could identify both known and previously undetected clusters of meningococcal disease.

## Methods

### Ethics Statement

The study was approved by the Johns Hopkins Bloomberg School of Public Health, University of Pittsburgh, Emory University, Georgia Department of Public Health, Vanderbilt University School of Medicine and Tennessee Department of Health institutional review boards. Institutional review board approval was not required from the California, Colorado, Connecticut, Minnesota, New York or Oregon Active Bacterial Core surveillance sites because meningococcal disease is a reportable condition and the activities fall under routine disease surveillance authority.

### Study Isolates and Determination of Serogroup

Invasive meningococcal study isolates were obtained through active surveillance during the period of January 1, 2000-December 31, 2005 from 9 Active Bacterial Core surveillance (ABCs) sites. ABCs, an active laboratory- and population-based surveillance program for invasive bacterial pathogens, is a core component of the Centers for Disease Control and Prevention (CDC) Emerging Infections Programs Network. [12] ABCs defines a case as isolation of *N. meningitidis* from a normally sterile site, such as blood or cerebrospinal fluid, in a resident of an ABCs surveillance area. [13], [14] The CDC case definition of a serogroup C *N. meningitidis* outbreak is ≥3 confirmed or probable cases in ≤3 months, resulting in a primary attack rate of ≥10 cases per 100,000 population among persons with a common organizational affiliation or who live in the same community. [4] Although the Advisory Committee on Immunization Practices (ACIP) definitions and guidelines for the control and prevention of meningococcal disease were initially developed for serogroup C meningitis, the same principles are relevant for the control of cases attributable to other vaccine-preventable *N. meningitidis* serogroups including A, Y and W-135 [4].

Participating ABCs sites included the following areas: California (three counties in the San Francisco Bay area), Colorado (5 counties in the Denver area), Connecticut, Georgia, Maryland, Minnesota, New York (7 counties in the Rochester area and 8 in the vicinity of Albany), Oregon, and Tennessee (11 urban counties in the Nashville, Memphis, Knoxville and Chattanooga areas). The population under surveillance in 2005 was approximately 38 million persons [15].

Laboratory work for this study was performed at the CDC and the University of Pittsburgh. Serogrouping, MLST, and OMP genotyping of *por*A VR1 and VR2, *por*B, and *fet*A VR were performed as previously described. [7], [11] Meningococcal clones were classified according to serogroup, ST, and OMP (*porA*, *porB*, and *fetA*) genotyping. To be considered as belonging to the same clone, isolates had to be indistinguishable based on the results of these assays.

### Geotemporal Cluster Analysis

The methodology of cluster detection using SaTScan version 9.1.1 (Information Management Services, Inc., Silver Spring, MD, and Martin Kulldorff, Boston, MA) is available on the Technical Documentation page of the developer’s web site (www.satscan.org/techdoc.html). [16]–[21] All analyses were conducted using a dedicated personal computer with 4 GB of RAM. Case data were aggregated to the census tract level and all non-singleton clones were assessed for geotemporal clustering using retrospective space-time analyses with a discrete Poisson probability model. Analyses tested the hypothesis that the expected number of cases in each census tract was proportional to its population size. The scan identified clusters with high rates of *N. meningitidis* indicative of non-random spatial and/or temporal distribution of cases.

Date of isolate culture was used to define the onset date of illness. The units of time precision and time aggregation for all analyses were days and months, respectively. The maximum temporal window size for all analyses was 50% of the study period (36 months), and the maximum spatial window 50% of the population in each ABCs site. We chose these wide windows to gain insight into the persistence of invasive meningococcal clones over space and time. Our study aimed to assess the geotemporal distribution of molecularly related clusters and, accordingly, the selection of this long temporal window allowed identification of clusters demonstrating a persistence of specific clones with durations exceeding those of classically defined meningococcal outbreaks. When the maximum window is set to 50% of the population, both small and large clusters can be identified. The statistical significance of each cluster was determined by 999 replications of Monte Carlo hypothesis testing and interpreted as significant when p≤0.05. Nine ABCs sites were analyzed independently. To detect possible clustering across contiguous ABCs sites, the following combined locations were also analyzed: (1) the census tracts comprising metropolitan Chattanooga, TN, and the state of Georgia; (2) the census tracts in the vicinity of Albany, NY, and the state of Connecticut. ABCs sites were asked to determine whether the clusters reported herein were identified at the time they occurred, and whether the current study failed to detect any previously known clusters.

### Population and Geographic Data

Census 2000 geographic boundary files for states, counties and census tracts participating in ABCs were obtained from the Census Cartographic Boundary Files Collection of the U.S. Census Bureau’s Geography Division. [22] Population and land area data for ABCs surveillance sites were retrieved from the U.S. Census Bureau Census 2000 Summary File (SF 1) 100-Percent Data Set. [23] Using ArcGIS version 9.3.1 (ESRI, Inc., Redlands, CA), the location of each case was geocoded using a perturbation algorithm. The perturbation distance was designed to be an inverse function of the population density in the respective census tract. The purpose of the perturbation is to de-identify the location of individual cases on a map. As a result, geocoded case locations may exhibit displacement into an adjacent census tract in figures. Analyses were conducted at the level of census tract with cases aggregated to their true tract of residence. Census tract population density (residents per km^2^) and attack rate (per 100,000 population) were calculated based on tract population and case count.

## Results

A total of 1,159 *N. meningitidis* isolates were obtained from cases meeting the ABCs case definition between January 1, 2000 and December 31, 2005. 1,062 (91.7%) of these cases were successfully geocoded and included. The case counts by ABCs site were California, 113 (10.6% of the total); Colorado, 49 (4.6%); Connecticut, 82 (7.7%); Georgia 163 (15.4%); Maryland, 117 (11.0%); Minnesota, 126 (11.9%); New York, 75 (7.1%); Oregon, 267 (25.1%); and Tennessee, 70 (6.6%). Isolates were characterized by molecular subtyping into 438 unique clones, 78 (17.8%) of which were non-singletons. Overall there were 702 cases attributable to non-singleton clones, accounting for 66.0% of all geocoded isolates. Molecular characteristics of the non-singletons, ranked in decreasing order of frequency, are shown in Table 1.

**Table 1:** Case counts of non-singleton meningococcal clones, ranked by decreasing frequency.

Clone ID	Serogroup	Clone (CC: ST: *porB*: *porA* VR1, *porA* VR2: *fetA*)	Count (% of total)	ABCs site(s) with clone
1	Y	23∶23∶3-36: P1.5-2,10-1: F4-1	142 (13.4)	CA,CO,CT,GA,MD,MN,NY,OR,TN
2	B	32∶32∶3-24: P1.7,16: F3-3	113 (10.6)	CA,CO,CT,GA,MD,MN,OR,TN
3	Y	23∶23∶2-55: P1.5-1,2-2: F5-8	59 (5.6)	CA,CO,CT,GA,MD,MN,NY,OR,TN
4	C	11∶11∶2-2: P1.5,2: F3-6	40 (3.8)	CA,CO,CT,GA,MD,MN,NY,OR,TN
5	B	32∶32∶3-24: P1.7,16-20: F3-3	27 (2.5)	CA,CO,GA,MD,MN,OR
6	C	11∶11∶2-2: P1.5,2: F1-30	22 (2.1)	CO,CT,GA,MD,MN,OR,TN
7	B	162∶162∶3-73: P1.22,14: F5-9	16 (1.5)	CA,GA,MD,MN,NY,TN
8	B	32∶32∶3-36: P1.7,16: F3-3	14 (1.3)	OR
9	C	11∶2962∶2-75: P1.5-1,10-4: F3-6	14 (1.3)	CA,GA,MD,NY,OR,TN
10	C	11∶11∶2-2: P1.22-1,14: F3-6	13 (1.2)	GA,MD,MN,NY
11	B	32∶3584∶3-1: P1.7,16: F3-3	10 (0.9)	OR
12	C	103∶2006∶2-110: P1.5-1,10-4: F3-9	10 (0.9)	MN
13	C	11∶11∶2-2: P1.5-1,10-8: F3-6	10 (0.9)	GA,MD,MN,NY
14	B	32∶32∶3-1: P1.7,16: F3-3	9 (0.8)	CT,GA,MD,NY,OR
15	C	11∶2962∶2-88: P1.5-1,10-4: F3-6	9 (0.8)	CO,GA,MD,MN,OR,TN
16	B	32∶32∶3-84: P1.7,16: F3-3	8 (0.8)	GA,TN
17	Y	23∶1625∶2-55: P1.5-1,2-2: F5-8	7 (0.7)	CT,GA,MD,MN,OR,TN
18	Y	23∶23∶3-36: P1.5,2: F4-1	7 (0.7)	CA,CT,MN,OR,TN
19	B	41/44∶136∶3-107: P1.17,16-3: F5-5	6 (0.6)	CA,CO,GA,NY,TN
20	C	11∶2961∶2-48: P1.5,2: F1-30	6 (0.6)	MD,NY
21	B	35∶35∶3-39: P1.22-1,14: F4-1	5 (0.5)	GA,MD,OR
22	B	41/44∶154∶3-1: P1.7-2,4: F1-5	5 (0.5)	CA,CT,OR
23	C	11∶11∶2-73: P1.5,2: F3-6	5 (0.5)	GA,MD,MN,NY
24	C	32∶32∶3-24: P1.7,16: F3-3	5 (0.5)	MN,OR
25	W-135	22∶22∶2-23: P1.18-1,3: F4-1	5 (0.5)	CO,MN,OR,TN
26	Y	167∶1624∶2-55: P1.5-1,10-4: F3-4	5 (0.5)	CT,GA,NY,OR
27	B	32∶32∶3-133: P1.7,16-20: F3-3	4 (0.4)	OR
28	B	41/44∶4682∶3-71: P1.22-1,14: F5-2	4 (0.4)	CO,OR
29	C	11∶11∶2-2: P1.5,2: F1-5	4 (0.4)	CA
30	C	11∶11∶2-2: P1.5-1,2-2: F3-6	4 (0.4)	GA,NY,OR
31	Y	23∶1625∶2-141: P1.5-1,2-2: F5-8	4 (0.4)	OR
32	Y	23∶3582∶2-55: P1.5-1,2-2: F5-8	4 (0.4)	OR
33	B	32∶32∶3-1: P1.7,16-20: F3-3	3 (0.3)	CT,MD
34	B	41/44∶136∶3-107: P1.17,16-23: F5-5	3 (0.3)	CA,GA,TN
35	B	41/44∶170∶3-138: P1.21,16: F1-5	3 (0.3)	OR
36	B	41/44∶42∶3-1: P1.7-2,4: F1-5	3 (0.3)	CA
37	B	41/44∶44∶3-38: P1.7-1,1: F1-7	3 (0.3)	NY,OR
38	B	41/44∶44∶3-45: P1.7-4,1: F1-7	3 (0.3)	OR
39	B	41/44∶5097∶3-1: P1.7-2,4: F1-5	3 (0.3)	TN
40	B	41/44∶5111∶3-45: P1.21,16: F1-7	3 (0.3)	CT
41	C	No CC:2048∶3-16: P1.5,2: F3-6	3 (0.3)	GA
42	C	11∶11∶2-2: P1.17,16-3: F3-6	3 (0.3)	MD
43	C	11∶11∶2-2: P1.5,2: F3-3	3 (0.3)	CA,OR
44	C	11∶11∶2-2: P1.5,2: F5-5	3 (0.3)	OR
45	C	11∶11∶2-2: P1.5-1,2: F5-36	3 (0.3)	MN
46	Y	23∶1621∶3-36: P1.5-2,10-1: F4-1	3 (0.3)	MD
47	B	No CC:2048∶3-16: P1.12-1,16-8: F3-6	2 (0.2)	CO,OR
48	B	No CC:2875∶2-136: porA Deletion: F4-1	2 (0.2)	GA
49	B	254∶254∶3-223: P1.19,15: F1-5	2 (0.2)	GA
50	B	269∶2974∶3-113: P1.7-2,13-1: F5-7	2 (0.2)	MD
51	B	32∶1364∶3-24: P1.7,16: F3-3	2 (0.2)	CA,OR
52	B	32∶32∶3-107: P1.22-1,14: F3-3	2 (0.2)	GA
53	B	32∶32∶3-1: P1.7,16-33: F3-3	2 (0.2)	NY,OR
54	B	32∶32∶3-24: P1.7,16: F1-7	2 (0.2)	OR
55	B	41/44∶318∶3-1: P1.7-2,4: F1-7	2 (0.2)	MN
56	B	41/44∶409∶3-82: P1.18-1,34-2: F1-5	2 (0.2)	CA,GA
57	B	41/44∶41∶3-172: P1.7-2,4: F1-5	2 (0.2)	GA,TN
58	B	41/44∶41∶3-1: P1.7-2,4: F1-5	2 (0.2)	CT
59	B	41/44∶437∶3-114: P1.22-1,14: F5-2	2 (0.2)	MD
60	B	41/44∶437∶3-71: P1.22-1,14: F5-2	2 (0.2)	GA
61	B	41/44∶43∶3-16: P1.19,15-1: F1-5	2 (0.2)	GA,NY
62	B	41/44∶4489∶3-1: P1.7-2,4: F1-5	2 (0.2)	GA
63	B	60∶60∶3-8: P1.22-1,14: F3-9	2 (0.2)	MD
64	C	103∶5837∶2-22: P1.17,16-3: F1-18	2 (0.2)	TN
65	C	11∶11∶2-2: P1.5,2: F3-1	2 (0.2)	MD
66	C	11∶11∶2-2: P1.5,2: F4-12	2 (0.2)	CA,OR
67	C	11∶11∶2-2: P1.5-2,10-2: F5-36	2 (0.2)	MN
68	C	11∶11∶2-85: P1.22-1,14: F3-6	2 (0.2)	MN
69	C	41/44∶41∶3-1: P1.7-2,4: F1-5	2 (0.2)	CA
70	C	8∶8∶2-3: P1.5,2: F5-8	2 (0.2)	MN
71	W-135	22∶1476∶2-23: P1.18-1,3: F4-1	2 (0.2)	CO,OR
72	W-135	22∶22∶2-109: P1.18-1,3: F4-1	2 (0.2)	CO
73	Y	22∶1265∶2-23: P1.18-1,3: F1-7	2 (0.2)	GA
74	Y	23∶183∶3-53: P1.5-2,10-2: F4-1	2 (0.2)	NY
75	Y	23∶23∶3-36: P1.5-2,10-12: F4-1	2 (0.2)	MD,TN
76	Y	23∶23∶3-36: P1.5-2,10-29: F4-1	2 (0.2)	GA,NY
77	Y	23∶23∶3-53: P1.5-2,10-2: F4-1	2 (0.2)	GA,NY
78	Y	23∶3587∶3-36: P1.5-2,10-2: F4-1	2 (0.2)	GA,OR

ST, sequence type; CC, clonal complex; VR, variable region; CA, California; CO, Colorado; CT, Connecticut; GA, Georgia; MD, Maryland; MN, Minnesota; NY, New York; OR, Oregon; TN, Tennessee.

Given the large number of distinct non-singleton clones identified (116 distinct site/clone parings, with 7 separate analysis files for each–one for each of the 6 study years, and a composite 2000–2005 file), parameterizing and executing the scans to generate the results reported herein was time and computationally intensive. Individual scans took anywhere from seconds to upwards of 24 hours.

**Figure 1:**
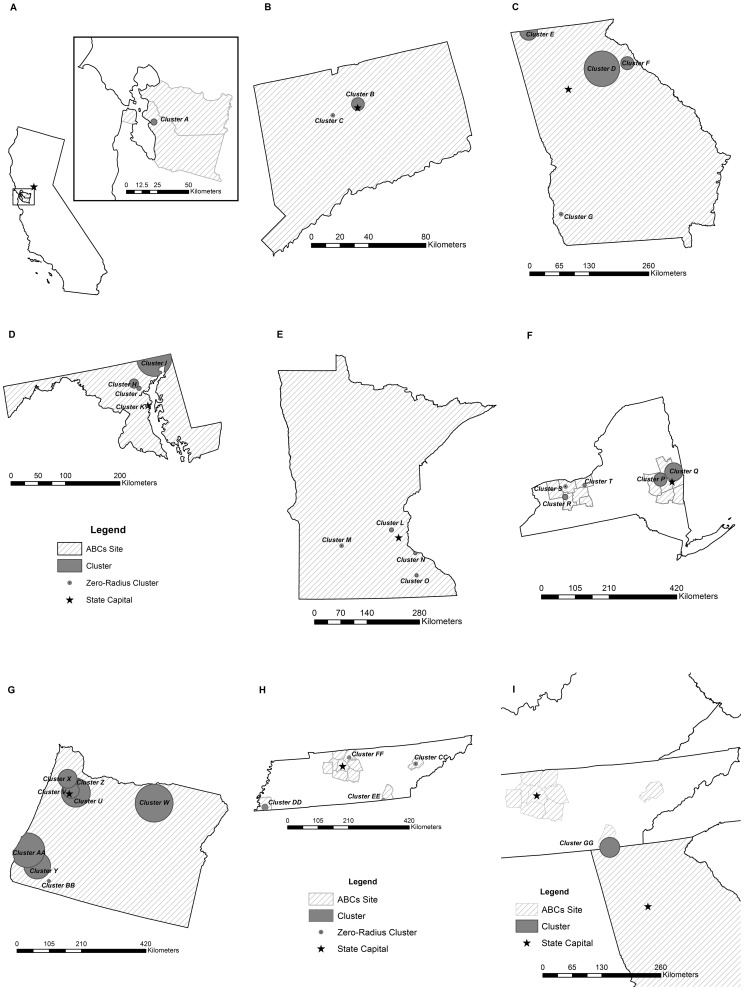
Meningococcal clusters, by Active Bacterial Core surveillance site, 2000–2005. (A) California with San Francisco Bay area inset; (B) Connecticut; (C) Georgia; (D) Maryland; (E) Minnesota; (F) New York; (G) Oregon; (H) Tennessee; (I) Georgia and Tennessee.

Thirty-two statistically significant clusters involving 107 cases (10.1% of all geocoded isolates) attributable to 23 distinct clones were identified (Table 2 and Figure 1) by independent analysis of each ABCs site. Clusters were identified in all sites except Colorado. Clusters ranged in duration from 1 day to 33 months. 19 clusters (59.4%) were composed of two cases and 13 (40.6%) included 3 or more cases. Incidence ranged from 0.7 to 57.8 per 100,000, with 10 clusters (31.3%) having an attack rate of ≥10 cases per 100,000 population over the cluster time period. Annualized incidence ranged from 1.1 to 693.6 cases per 100,000 population. The range of cluster radii was 0 to 61.7 km. Five clusters (15.6%) had radius of 0 km, indicating that the involved cases occurred within a single census tract. Three of the five zero-radius clusters (Clusters C, G and M) were previously identified by the respective ABCs sites, and all were noted to be case pairs among household members. Two zero-radius clusters (Clusters S and CC), also comprised of case pairs, were previously unidentified. No purely temporal clusters (those encompassing an entire ABCs surveillance area) or purely spatial clusters (those with a temporal window spanning the entire 72 month study period) were identified.

**Table 2:** Characteristics of statistically significant meningococcal disease clusters.

Cluster ID	Clone ID^A^	Serogroup	Site	First Case	Last Case	Radius (km)	Population	No. Cases	Attack Rate (per 100,000)	Expected Cases	Observed/Expected	Annualized Incidence (per 100,000)	p-value
A	1	Y	CA	1/9/2000	3/30/2000	2.5	76,377	4	5.2	0.021	190.5	20.9	0.003
B	3	Y	CT	11/26/2001	4/17/2002	4.5	105,973	4	3.8	0.049	81.6	9.1	0.027
C	3	Y	CT	2/19/2005	3/15/2005	0.0^B^	3561	2	56.2	0.00053	3773.6	674.0	0.022
D	1	Y	GA	2/27/2000	9/15/2001	38.8	399,878	9	2.3	0.53	17.0	1.4	0.003
E	16	B	GA	1/22/2005	7/7/2005	19.3	95,677	4	4.2	0.0045	888.9	8.4	0.001
F	48	B	GA	11/24/2000	9/16/2001	14.3	20,511	2	9.8	0.00076	2631.6	11.7	0.014
G	62	B	GA	6/11/2002	6/11/2002	0.0^B^	3956	2	50.6	0.000013	153,846.2	606.7	0.001
H	1	Y	MD	12/21/2000	2/28/2001	8.5	361,669	5	1.4	0.062	80.6	5.5	0.004
I	10	C	MD	3/26/2001	6/19/2001	31.7	262,096	2	0.8	0.0055	363.6	3.1	0.049
J	46	Y	MD	3/6/2000	3/7/2000	3.9	169,845	2	1.2	0.0014	1428.6	14.1	0.023
K	59	B	MD	2/8/2001	4/2/2001	1.8	9588	2	20.9	0.00015	13,333.3	125.2	0.003
L	4	C	MN	8/10/2000	8/19/2000	5.5	111,010	2	1.8	0.0013	1538.5	21.6	0.038
M	13	C	MN	10/30/2002	11/1/2002	0.0^B^	3460	2	57.8	0.00012	16,666.7	693.6	0.004
N	67	C	MN	8/21/2002	3/15/2003	4.4	16,616	2	12.0	0.00075	2666.7	20.6	0.011
O	70	C	MN	3/31/2002	4/17/2002	4.4	23,597	2	8.5	0.00027	7407.4	101.7	0.005
P	9	C	NY	12/3/2002	12/11/2002	20.4	138,884	2	1.4	0.0019	1052.6	17.3	0.010
Q	10	C	NY	2/4/2001	8/1/2001	27.9	382,319	4	1.0	0.070	57.1	2.1	0.007
R	20	C	NY	11/3/2001	5/14/2002	8.3	7818	2	25.6	0.0018	1111.1	43.9	0.027
S	26	Y	NY	4/8/2000	12/12/2002	0.0^B^	8549	2	23.4	0.0037	540.5	8.5	0.017
T	37	B	NY	9/25/2001	2/9/2004	5.2	10,547	2	19.0	0.0042	476.2	7.8	0.022
U	1	Y	OR	8/10/2000	11/2/2000	46.9	717,344	5	0.7	0.15	33.3	2.8	0.033
V	2	B	OR	12/3/2000	4/18/2001	25.8	300,817	10	3.3	0.59	16.9	8.0	0.001
W	4	C	OR	9/21/2002	10/11/2002	61.7	33,851	3	8.9	0.00083	3614.5	106.3	0.001
X	8	B	OR	1/24/2000	12/31/2001	30.6	138,831	11	7.9	0.19	57.9	4.0	0.001
Y	14	B	OR	2/13/2001	2/23/2001	43.4	92,926	2	2.2	0.0014	1428.6	25.8	0.025
Z	24	C	OR	12/30/2004	1/2/2005	2.3	20,767	2	9.6	0.00034	5882.4	115.6	0.006
AA	35	B	OR	1/25/2000	7/31/2000	56.2	90,871	3	3.3	0.0077	389.6	5.7	0.003
BB	54	B	OR	3/10/2004	5/3/2004	4.6	49,121	2	4.1	0.0012	1666.7	24.4	0.012
CC	1	Y	TN	6/28/2001	9/16/2001	0.0^B^	3863	2	51.8	0.0011	1818.2	207.1	0.032
DD	9	C	TN	9/25/2001	8/22/2002	11.0	397,456	4	1.0	0.094	42.6	1.1	0.012
EE	16	B	TN	10/9/2005	10/20/2005	6.1	24670	2	8.1	0.00050	4000.0	97.3	0.011
FF	39	B	TN	4/22/2003	3/2/2005	6.6	25,579	3	11.7	0.0091	329.7	6.1	0.004
GG	16	B	GA/TN	1/22/2005	10/20/2005	21.9	341,092	7	2.1	0.045	155.6	2.7	0.001

Table 1 for detailed information on the molecular characteristics of each clone.^A^ See

^B^ Case data were aggregated to the level of census tract for these analyses. A radius of 0 km indicates that all cases in that cluster occurred within the same census tract.

In analyses of contiguous ABCs sites, one cluster (cluster GG) caused by clone 16 included 7 cases in metropolitan Chattanooga, TN and northwestern Georgia (radius = 21.9 km) that occurred between January 22, 2005 and October 20, 2005 (Figure 1). In single-site analyses, a 4-case cluster of Clone 16 (cluster E, *p = *0.001, radius = 19.3 km) was detected in Georgia and a 2-case cluster in Tennessee (cluster EE, *p = *0.011, radius = 6.1 km). The cross-site analysis captured an additional case in Tennessee not associated with the independently identified cluster in that state.

Eleven clusters (34.4% of all clusters) had previously been identified by the ABCs sites, while 21 clusters (65.6%) had not been identified. Cluster GG, which spanned the Georgia-Tennessee border, was previously identified by local public health practitioners. A cluster comprised of 2 cases occurring 1 week apart among students at the same university was reported by Connecticut but not detected in our analyses. These isolates were identical by OMP genotyping but differed at a single MLST locus (ST-1374 versus ST-40).

Three serogroups were represented among the clusters: B (n = 12 clusters, 45 cases), C (n = 11 clusters, 27 cases) and Y (n = 9 clusters, 35 cases), indicating that 20 clusters (62.5%) were caused by serogroups represented in meningococcal vaccines that are available in the U.S. Although seven cases attributable to two distinct serogroup W-135 clones were reported, no significant serogroup W-135 *N. meningitidis* clusters were identified. Eleven distinct serogroup B clones were responsible for 12 significant clusters (37.5% of all clusters) in 5 states: Georgia (3), Maryland (1), New York (1), Oregon (5), Tennessee (2), and an additional cross-site cluster in Georgia/Tennessee. No cluster attributable to a vaccine-preventable serogroup met the CDC definition of an outbreak warranting consideration of vaccination for disease control, and our analyses did not fail to detect any clusters that prompted local health officials to consider vaccination as a control strategy.

Sensitivity analyses were conducted to assess the impact of alternate scanning window parameterizations. Spatial and temporal windows were varied from 25–50% and 25–90%, respectively. The clusters detected differed negligibly for each permutation of settings, so we elected to present the results for an intermediate set of parameterizations with spatial and temporal windows each set at 50%.

## Discussion

We identified 32 meningococcal case clusters with non-random spatial and temporal distribution, 21 (65.6%) of which had not been previously identified. To our knowledge, this is the first study of the geotemporal distribution of meningococcal clones causing invasive disease in the United States. Our analyses highlight the large diversity of circulating meningococcal clones in the U.S. and the fact that most invasive meningococcal disease is caused by a limited number of MLST-defined meningococcal lineages. Importantly, 10.1% of cases belonged to a molecularly identified cluster, which is higher than the proportion of cases identified in outbreaks using traditional epidemiologic methods. [3] All five clusters with a radius of 0 km (indicating that both cases in the pair occurred in the same census tract) were associated with case pairs. The attack rate and projected annualized incidence rate in the affected census tracts ranged from 23.4 to 57.8 and 8.5 to 693.6 cases per 100,000 population, respectively. This highly non-random spatial and temporal distribution of cases underscores the importance of assessing the geotemporal distribution of as few as 2 isolates of the same clone. We did not detect any serogroup C clusters that met the CDC definition of an outbreak warranting consideration of vaccination. Assuming the ACIP serogroup C outbreak definition thresholds, no clusters of serogroup Y disease were detected that would prompt consideration of vaccination for disease control. [4] This lack of geographically expansive clusters comprised of large numbers of cases may be, in part, a reflection of the historically low incidence of meningococcal disease in the United States. [2] During the study period the national projection for the annual incidence of meningococcal disease in the U.S. decreased from 0.8 cases per 100,000 population (year 2000) to 0.35 cases per 100,000 population (2005), with a nadir of 0.31 cases per 100,000 in 2004 [24]–[29].

The finding of a cluster spanning the Georgia-Tennessee border underscores the importance of looking across jurisdictional boundaries and the use of a common definition of clone. Cross-border spread has also been implicated in the increased incidence of cases seen in the Aachen region of Germany and neighboring Netherlands. [30] This edge effect was thought to explain the excess number of districts surrounding Aachen which had incidence rates higher than could be explained by complex space-time conditional intensity modeling of Germany alone. [31] In a study of 3,979 cases of meningococcal disease occurring from 2002–2008 in Niger, 15 clusters were identified. [32] Clusters ranged in size from 9 to 558 cases, and exhibited a geographic predominance in southeastern Niger. Movement across the border between Niger and Nigeria in this region is common and represents another example of this edge effect.

A previous spatiotemporal analysis of invasive meningococcal disease in Germany used a molecular subtyping scheme similar to ours with the exception that it did not include *porB* genotyping. [8] This approach, applied to 1,616 cases, resulted in the identification of 383 unique clones. 4.2% of cases were involved in a cluster, and 76.9% of clusters involved only 2 patients. Although the proportion of cases assigned to a cluster was lower in that study than ours (10.1%), a similarly high number of detected clusters included 2 patients (60% in our study).

Clone 2, a serogroup B strain, predominated in Oregon where 97 cases (85.8% of all Clone 2 isolates) were identified during the study period. These isolates represented 36.3% of all invasive meningococcal cases in Oregon from 2000–2005. As reported previously, the Oregon clone also caused disease in seven other ABCs sites, but it was not implicated in any statistically significant clusters in other states, confirming previous reports of its unique epidemiologic behavior in Oregon [11].

The SaTScan methodology has several advantages for the study of clustering among cases of invasive meningococcal disease. The software is highly flexible and can be used for spatial, temporal or space-time scan statistics, in either a retrospective or prospective fashion. Scan parameters, including spatial and temporal windows can be tailored to assess for specific types of endemic or epidemic patterns of activity. We purposely set relatively large windows with the goal of detecting patterns of transmission and disease beyond what would be defined as a community outbreak. [4] Retrospective analyses aid the confirmation of previously detected clusters. Most importantly, this methodology can be used to detect associations between cases without a previously identified epidemiological link. Prospective scans allow for the early detection of related cases and may signal persistence of a clone in space or time.

SaTScan allows the user to define clone characteristics, so parameters can be tailored to complement the level of molecular typing available. In this study, we used a highly conservative definition of a clone, namely, identification by MLST and genotyping of 3 OMP antigens. More clusters would have been detected if *porB* genotyping results had been restricted to the level of class 2 or 3 genotypes. If we had collapsed porB into class 2 or 3, we would have identified 352 unique clones (a 19.6% decrease), including 80 non-singletons. Using this classification, 790 cases would be attributable to a non-singleton clone, accounting for 74.4% of geocoded isolates. With *por*B collapsed into class 2 or 3 and all other scan parameters unchanged, we would have detected 50 clusters, as opposed to the 32 detected with the stricter definition used in this study. Consistent with the 2-case cluster reported by Connecticut, in which epidemiologically related isolates were indistinguishable except for a single locus difference by MLST, a less-conservative definition of clone, while leading to the detection of more clusters, would also have likely falsely clustered unrelated cases. Regardless, the specific SaTScan settings and definition of a clone can be tailored to the specific setting in which this methodology is employed.

In summary, the SaTScan methodology is a flexible and practical tool for the surveillance and analysis of meningococcal disease. Geotemporal scan software, in conjunction with molecular subtyping, can be used to study patterns of infectious disease transmission, identify previously undetected clusters, and improve understanding of the epidemiology of circulating molecular clones.
